# Pharyngeal Histoplasmosis Presenting as a Tumor Mass in an Immunocompetent Host

**DOI:** 10.4103/0974-777X.59256

**Published:** 2010

**Authors:** Fouzia Siraj, Varsha Manucha

**Affiliations:** *Department of Pathology, Sir Ganga Ram Hospital, New Delhi - 110 060, India*

Sir,

Histoplasmosis is an infrequently reported disease in India, and we have come across sporadic case reports from various parts of the country. We would like to share details of a case of oropharyngeal histoplasmosis that we recently came across in our practice, because of its unique presentation and its histopathologic appearance. The patient was a 62-year-old immunocompetent man who presented with a mass in the pharynx. His chief complaints were progressively worsening difficulty in swallowing and alteration of voice of 4 months duration. The patient was a known diabetic. Findings from his baseline investigations, including X-ray chest, were within normal limits. Indirect laryngoscopy revealed presence of a mass in the left posterior pharyngeal wall, extending up to the base of tongue and involving the epiglottis. The lower limit of the mass was not visualized. A clinical diagnosis of a possible malignant lesion in the pharynx was considered. A punch biopsy was performed. Histopathological examination revealed a highly cellular lesion composed of sheets of polygonal cells with eosinophilic granular cytoplasm, separated by small blood vessels [Figure 1]. No mitotic activity or necrosis was seen. Initial impression was that of a benign tumor. However, on careful inspection, intracellular inclusions with peripheral halo were seen within the cells at the periphery of the lesion [Figure 2]. Sprinkling by lymphocytes and plasma cells was noted. No feature of active inflammation, including ulceration or granulation tissue, was seen. No granulomas were seen. A Gomori methamine silver stain highlighted small oval yeast forms with narrow-base budding [Figure 2]. Immunostain for CD68 highlighted histiocytes. A diagnosis of oropharyngeal mycosis with fungal forms morphologically consistent with histoplasma was rendered, and a microbiological evaluation was suggested. On further investigations, it was found that the patient was a construction engineer with a history of traveling within and outside the country. He did not have any features of chronic lung disease or enlarged lymph nodes that are often described with oral histoplasmosis.

**Figure 1:**
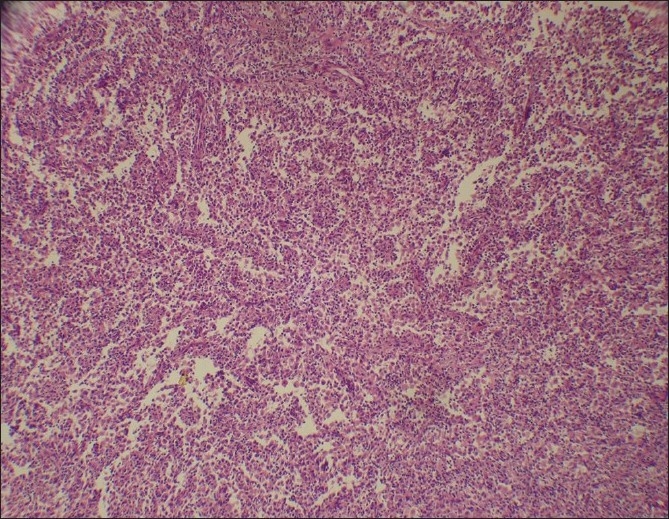
Highly cellular lesion composed of sheets of histiocytes (H and E, ×200)

**Figure 2:**
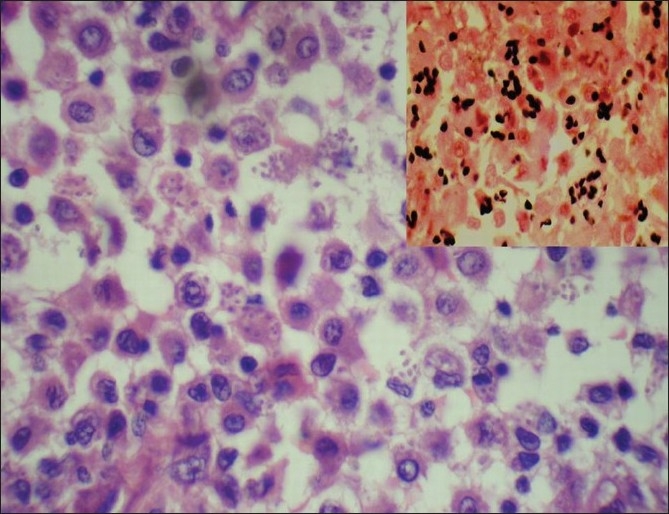
Intracellular inclusions with peripheral halo and narrow-base budding (H and E, ×400). Inset: Gomori methamine silver stain (×1000)

Histoplasmosis in immunocompetent host usually presents as a mild or clinically insignificant respiratory disease. Mucus membrane lesions are more common in indolent cases and are found in up to two thirds of the chronic cases.[1] Most of these lesions are ulcerated and are commonly accompanied by hepatosplenomegaly and adrenal insufficiency.[1,2] Presentation as an isolated mass lesion in the pharynx is rare and can be clinically misdiagnosed as a tumor.

The available information on the disease in India is largely based upon sporadic case reports and so the endemic zones of histoplasmosis have not been delineated; consequently, the disease should not be suspected only on the basis of the patient's origin.

We present this case because histoplasmosis can pose a diagnostic dilemma to both the clinicians and pathologists. Oral histoplasmosis is difficult to diagnose clinically as it can resemble other more common clinical entities such as carcinoma (as in our case), syphilis, sarcoidosis, lymphoma and tuberculosis. A pathologist should suspect or look for histoplasmosis when an isolated population of macrophages is seen in absence of other inflammatory cells or granulomas.

## References

[1] Randhawa HS, Khan ZU (1994). Histoplasmosis in India: Current status. Indian J Chest Dis Allied Sci.

[2] Padhye AA, Pathak AA, Katkar VJ, Hazare VK, Kaufman L (1994). Oral histoplasmosis in India: A case report and an overview of cases reported during 1968-92. J Med Vet Mycol.

